# SLiMFinder: A Probabilistic Method for Identifying Over-Represented, Convergently Evolved, Short Linear Motifs in Proteins

**DOI:** 10.1371/journal.pone.0000967

**Published:** 2007-10-03

**Authors:** Richard J. Edwards, Norman E. Davey, Denis C. Shields

**Affiliations:** 1 University College Dublin Complex and Adaptive Systems Laboratory, University College Dublin Conway Institute of Biomolecular and Biomedical Sciences, University College Dublin, Dublin, Ireland; 2 School of Biological Sciences, University of Southampton, Southampton, United Kingdom; Tata Institute of Fundamental Research, India

## Abstract

**Background:**

Short linear motifs (SLiMs) in proteins are functional microdomains of fundamental importance in many biological systems. SLiMs typically consist of a 3 to 10 amino acid stretch of the primary protein sequence, of which as few as two sites may be important for activity, making identification of novel SLiMs extremely difficult. In particular, it can be very difficult to distinguish a randomly recurring “motif” from a truly over-represented one. Incorporating ambiguous amino acid positions and/or variable-length wildcard spacers between defined residues further complicates the matter.

**Methodology/Principal Findings:**

In this paper we present two algorithms. SLiMBuild identifies convergently evolved, short motifs in a dataset of proteins. Motifs are built by combining dimers into longer patterns, retaining only those motifs occurring in a sufficient number of unrelated proteins. Motifs with fixed amino acid positions are identified and then combined to incorporate amino acid ambiguity and variable-length wildcard spacers. The algorithm is computationally efficient compared to alternatives, particularly when datasets include homologous proteins, and provides great flexibility in the nature of motifs returned. The SLiMChance algorithm estimates the probability of returned motifs arising by chance, correcting for the size and composition of the dataset, and assigns a significance value to each motif. These algorithms are implemented in a software package, SLiMFinder. SLiMFinder default settings identify known SLiMs with 100% specificity, and have a low false discovery rate on random test data.

**Conclusions/Significance:**

The efficiency of SLiMBuild and low false discovery rate of SLiMChance make SLiMFinder highly suited to high throughput motif discovery and individual high quality analyses alike. Examples of such analyses on real biological data, and how SLiMFinder results can help direct future discoveries, are provided. SLiMFinder is freely available for download under a GNU license from http://bioinformatics.ucd.ie/shields/software/slimfinder/.

## Introduction

Protein-protein interactions are of fundamental importance in biology. Although many well-characterised interactions are mediated by large domain-domain interfaces, it is estimated that 15%–40% of interactions may be mediated by a short, linear motif (SLiM) in one of the binding partners [1], [2]. Because of their short and degenerate nature, new SLiMs are hard to identify and much of what we know about them stems from a few well-characterised examples (*e.g.* SH2-domain binding motifs [3]). The Eukaryotic Linear Motif (ELM) database has annotated examples for over sixty known motifs [4] and large-scale analyses of interaction datasets suggest that there are hundreds yet to be discovered [5].

SLiM-mediated interactions are often transient, with quite low affinity for their binding partners, and it has been suggested that they exhibit considerable evolutionary plasticity [6]. Indeed, existing methods for identifying new SLiMs [7], [8] explicitly invoke a model of convergent evolution to identify over-represented sequence patterns. These methods, however, rely on an initial motif discovery phase using generic pattern-finding TEIRESIAS software [9], which returns all shared patterns regardless of evolutionary relationships and with only crude length and complexity control. As a result, a lot of post-processing of returned motifs is required. Furthermore, TEIRESIAS offers only limited ambiguity capabilities and no options for returning variable length wildcard spacers, such as seen in the Cyclin recognition site ([RK].L.{0,1}[FYLIVMP]) [4]. Here we present SLiMBuild, which is a novel algorithm explicitly designed to identify SLiMs that are shared by unrelated proteins (as identified by BLAST [10]). SLiMBuild constructs motifs by combining dimers into longer patterns before efficiently incorporating amino acid degeneracy and/or variable length wildcards by adding variants that (a) occur in the desired number of unrelated proteins, and (b) increase the total number of unrelated proteins in which the ambiguous motif occurs.

Identifying recurring motifs is only part of the challenge. Because of their relative simplicity, short motifs are expected to occur in multiple unrelated proteins by chance. To account for this, SLiM discovery tools attempt to attach a score that indicates how unlikely a given motif is compared to other motifs in a dataset, either through an explicit heuristic [8] or by an empirical estimate [7]. The SLiMChance algorithm we present here improves on these scores by making a crude but effective adjustment of motif probabilities by considering the total number of motifs in the motif-space considered by SLiMBuild. This allows the attachment of a significance value to returned motifs, which returns known motifs with a very high specificity from benchmark datasets of known eukaryotic motifs.

Both SLiMBuild and SLiMChance are implemented in a combined software package called SLiMFinder, which is freely available for academic use. SLiMFinder implements a number of input and output options that are described elsewhere (see http://bioinformatics.ucd.ie/shields/software/slimfinder/).

## Methods

The term “motif” can be used in a number of different contexts with different meanings. In this paper, we use motif to mean a short, linear motif (SLiM) in a protein. In biology, SLiMs are functional microdomains with three main properties:

1. *Short*–generally less than 10aa long with five or less defined residues.
2. *Linear*–comprised of adjacent amino acids in a protein's primary sequence. While three-dimensional conformation may be important for function, it is not necessary for definition.
3. *Motif*–a defined sequence pattern, which is necessary for function, recurs in the relevant proteins.

For simplicity, we use “SLiM” in this paper to describe a true functional motif with these properties, and “motif” to describe SLiM-like sequence patterns that may be functional or may simply be chance occurrences. SLiMs comprise of a number of defined amino acid positions, often separated by a number of wildcards, which may be any amino acid (Figure S1). Defined positions may be fixed, in which case only one species of amino acid is permitted at that position, or ambiguous, in which case multiple different amino acids may occupy that site and still result in a functional SLiM.

### Overview of SLiMFinder algorithms

**Figure 1:**
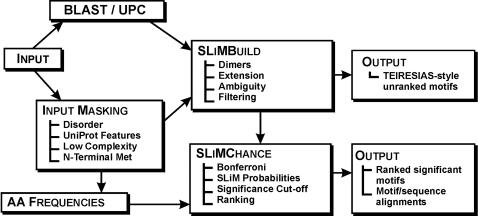
Overview of SLiMFinder. An input dataset is first clustered into unrelated protein clusters (UPC) using a treatment of BLAST results to identify evolutionary relationships. The dataset is also masked according to user choices, masking out predicted ordered regions, selected UniProt features, low complexity regions and/or N-terminal methionines. This (masked) dataset is then processed by the SLiMBuild algorithm to identify motifs that are shared by unrelated proteins. A TEIRESIAS-style output of all motifs can be produced at this point. Amino acid frequencies are calculated for each cluster of unrelated proteins, either before or after masking, and may be retained as cluster-specific frequencies or averaged over all clusters. Alternatively, amino acid frequencies may be given from an external source. These frequencies are combined with data from SLiMBuild on the motif composition of the dataset and processed by the SLiMChance algorithm, which identifies significantly over-represented motifs. These motifs and additional dataset information are then output into results files.

SLiMFinder is explicitly designed to look for shared motifs in regions of interest of unrelated proteins. To this end, evolutionary relationships must first be established using BLAST, before the main SLiMBuild Algorithm identifies shared motifs between unrelated proteins, masking out unwanted residues as required (Figure 1). The SLiMChance algorithm then assesses the motifs for statistically unlikely over-representation and significant motifs (putative SLiMs) are output (Figure 1). SLiMFinder recognises a number of input formats, although UniProt or Fasta format are recommended. Batch running of multiple datasets is also fully supported.

### Establishment of Evolutionary Relationships

SLiMFinder finds motifs that are shared by different “Unrelated Protein Clusters” (UPCs). Each UPC is a group of proteins that are not related to any proteins in the dataset outside of their own UPC. BLAST [10] is first used to identify which proteins are related to which other proteins. Each protein is grouped with all its BLAST hits and then iteratively grouped with *their* BLAST hits until no more sequences are added to the UPC. Each UPC therefore has the following characteristics:

1. Every protein in a UPC has a BLAST-detectable relationship with at least one other member of the UPC.
2. Every protein in a UPC can be linked to every other protein in the UPC via BLAST-detectable relationships, though sometimes this must go through one or more intermediate proteins.
3. None of the proteins within a UPC has a BLAST-detectable relationship with any of the proteins in another UPC.

By default, a BLAST e-value of 10^−4^ is used and the complexity filter is on. These parameters may be changed by the user.

### Input sequence masking

SLiMFinder offers a number of input masking options, which can be useful for restricting analyses to particular parts of the proteins in the dataset. These include IUPRED [11] disorder prediction, UniProt features and low complexity regions. SLiMFinder masking is performed after UPC definition and therefore masking will not affect the UP relationships between sequences. Full details of the masking options are available at the SLiMFinder website.

### SLiMBuild Construction of Motifs

SLiMBuild uses five basic sets of parameters for generating motifs from the dataset:

1. *w,* the maximum number of wildcard positions allowed between any adjacent pair of defined positions.
2. The maximum number of defined positions. (Sometimes referred to as the “length” of the motif, although the “true length” of a SLiM would include both defined and wildcard positions.)
3. *s*, the minimum support for the motif, i.e. the number of unrelated proteins that motif occurs in.
4. Ambiguity options, including an equivalency file of allowed ambiguities.
5. An optional minimum variant support, *v*, used in extending ambiguity.

**Figure 2:**
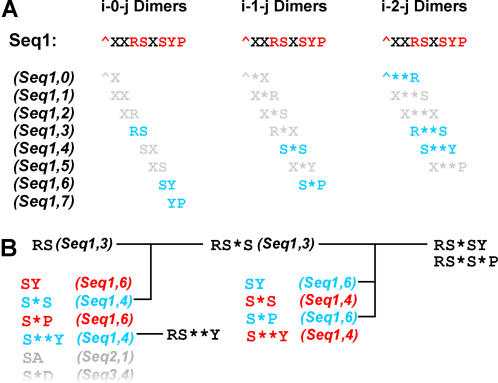
SLiMBuild construction of motifs. A. Dimer construction. For each position in a sequence, each possible wildcard length x is used to find possible “i-x-j” dimers. Dimers containing masked (“X”) residues are ignored (greyed dimers). Note that the n-terminal “^” marker is treated as any other amino acid. B. Motif extension. Longer SLiMs are constructed during the SLiMBuild process by matching the occurrences of shorter SLiMs with the relevant i-x-j dimers. At each stage, only SLiMs with sufficient unrelated protein support are retained, making the algorithm very efficient.

**Figure 3:**
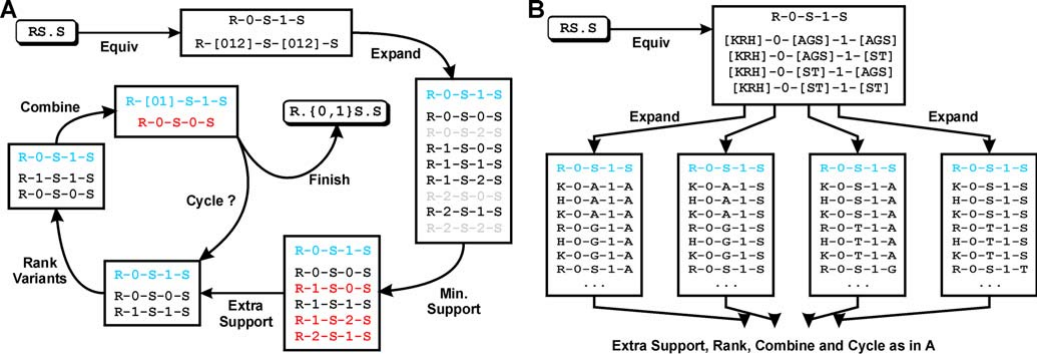
SLiMBuild Ambiguity. A. Wildcard ambiguity. Ambiguity is added in a multi-stage process. First, the motif is broken up into its component parts, consisting of alternate defined and wildcard positions. These are then replaced by the appropriate equivalency group, which in the case of wildcards is the full range of wildcard lengths from 0 up to the maximum length allowed. These equivalencies are then expanded to all possible variants. Any variants that do not themselves meet the minimum support requirement used previously for motif extension are not considered (shown in grey). Variants are only combined when the UPC support for the ambiguous motif is greater than for the individual variants. Variants that would not increase the UPC support of the original motif are therefore also removed (shown in red). The remaining variants are ranked (see text) and the best variant combined with the original motif (blue). The remaining variants are re-assessed for increasing UPC support and any failing to do so are again removed. If any remain, the ranking and combining cycle repeats. If not, the finished degenerate motif is returned. B. Amino acid ambiguities. These are handled in the same way as wildcard ambiguities, except that this time equivalencies are defined by the given equivalency list. If a given amino acid belongs to multiple equivalency groups, such as serine ([AGS] and [ST]) then all possible combinations of these equivalency groups (four in this case) are considered separately, thus multiple ambiguous SLiMs can potentially be produced. (Expansion of these combinations has been truncated in the figure.)

Motifs are constructed by first identifying all possible “*i-x-j* dimers”, which consist of two amino acids *i* and *j* separated by *x* wildcards, up to the maximum allowed value, *w* (Figure 2A). Motifs are then extended by joining appropriate dimers together (Figure 2B). Finally, SLiMBuild incorporates ambiguity into the motifs (Figure 3).

### SLiMBuild dimer construction

Dimers are constructed simply by taking each position *i* of each protein in turn to define the first amino acid, *a_i_*. Each wildcard length *x* from 0 to *W*, where *W* is the maximum wildcard length is then taken in turn and used to define the second amino acid in the dimer, *a_j_* where *j* = *i*+*x*. If *a_i_* or *a_j_* are masked (an ‘X’) then that dimer is rejected, else the dimer is added to the stored list, along with information on the protein and position *i* of its occurrence (Figure 2A). Symbols representing N- and C-termini (^ and $) are added to each sequence prior to dimer construction and thereon considered as additional amino acids. After all dimers have been found in all sequences, any with a support below the minimum support threshold are removed. (For a motif to exceed a given support, each of its component dimers must also exceed that support.)

### SLiMBuild motif extension

Motifs are extended by concatenating i-x-j dimers (Figure 2B). For each dimer *a_i_x_1_a_j_* all *a_z_x_2_a_k_* dimers are examined, where *a_z_* = *a_j_* (*k* = *z*+*x_2_*, *a_z_* and *a_k_* are amino acids at positions *z* and *k*). Where the two dimers have occurrences in the same protein and *z* = *j*, the two dimers are compiled to make a single *a_i_x_1_a_j_x_2_a_k_* trimer. If this trimer occurs in *s* or more unrelated sequences, it is retained and extended in the same way to make 4mers. This continues until the maximum motif length is reached (length 5 by default) or until there are no more motifs with the desired support to extend.

### SLiMBuild ambiguity

SLiMBuild considers two types of ambiguity: amino acid degeneracy at a given position, and flexible length wildcard “gaps”. A similar logic is applied in considering both these forms of ambiguity by carefully combining appropriate motifs generated during SLiMBuild extension. Each fixed motif is considered in turn as a seed for adding ambiguity in terms of degenerate non-wildcard positions and/or flexible wildcard lengths (Figure 3). Ambiguity is considered in three phases: wildcards only, amino acids only and combined wildcard and amino acid degeneracy. (Combined ambiguity can be computationally intensive and is switched off by default.)

In each case, the motif being considered is broken down into individual elements, consisting of alternate amino acids and/or wildcard lengths. Each element is then replaced by its “equivalencies”. For wildcards, this consists of single wildcard equivalency “01..*W*”, where *W* is the maximum wildcard length allowed; *e.g.* for the default maximum wildcard length of 2, the wildcard equivalencies are 0, 1 and 2, and a variable length gap of 1 or 2 is represented by the equivalency [12]. (Figure 3A). For amino acid positions, SLiMFinder makes use of an “Equivalency list” for ambiguity in a similar way to TEIRESIAS, although the actual application of this file is quite different. This equivalency list contains a number of amino acid groups that may be substituted in degenerate positions; *e.g.* KR would allow for [KR] degeneracy, while FYW, would facilitate [FY], [YW], [FW] and [FYW]. A single amino acid can have multiple equivalency groups, which are analysed separately. *E.g.* AGS,ST would permit serine [AS], [GS], [AGS] and [ST], but not [AGST]. Where multiple equivalency groups exist for one or more amino acids in a SLiM, all possible combinations of equivalency group are considered (Figure 3B).

The idea of ambiguity is to try to increase the coverage within a dataset for a given motif. This is achieved by adding ambiguity that increases support (no. of unrelated proteins) for the motif. Thus, returned motifs need to have been initially seeded by a non-ambiguous motif (with lower support) before it is extended to consider ambiguity. For each ambiguity combination, all possible variants (excluding the original motif) are then considered. *E.g.* [KR]-0-[ST]-1-P yields variant motifs K0S1P, R0S1P, K0T1P and R0T1P, the second of which is ignored as it is the original motif. Any variants that do not meet the minimum support requirement are also rejected. Remaining variants are then ranked according to the following criteria:

1. Number of “new” UP clusters. (The number of UPCs in which the variant is found but the original motif is not.) If the variant provides no new UPCs then it is rejected.
2. Total (UPC) support for the variant, if tied for 1.
3. Total number of occurrences for the variant (in different sequences, regardless of homology relationships), if tied for 1&2.
4. If tied for 1–3, the variant that is most unlikely, given the amino acid frequencies of the whole dataset, is ranked higher.

The top-ranked variant is retained and its UPCs added to those of the original motif. The ranking is then repeated using this new UP support, *i.e.* further variants are not added if their “extra” support has already been provided by previous variants. This continues until all variants have been retained, or rejected (Figure 3). Finally, retained variants are combined to make an ambiguous motif. *E.g.* if R0T1P had been retained then it would be combined with the original R0S1P SLiM to make R0[ST]1P (R[ST].P). In the case of flexible wildcards, the minimum and maximum length variants retained are used. *i.e.* R0S1P+R2S1P = R[02]S1P (R.{0,2}S.P). Note that because different equivalency combinations are examined separately, one SLiM may spawn several ambiguous motifs (*e.g.* R[ST].P and R[AGS].P) but only one ambiguity will be produced per equivalency group (*i.e.* R[AS].P and R[AGS].P will not both be produced using a single AGS equivalency group). Note also that each variant must itself meet the minimum (UPC) support criteria, so only recurring variants are combined.

### SLiMChance motif probability estimation

The SLiMChance algorithm attaches a significance value to motifs returned by SLiMBuild by first calculating the probability of seeing that specific motif in at least as many unrelated proteins as observed, and then adjusting this probability to take into consideration the total motif space searched by SLiMBuild.

### SLiMChance probabilities per UPC

SLiMChance first calculates the probability of seeing each motif in each UPC, given its amino acid composition and i-x-j dimer frequencies. This probability is calculated using the binomial distribution and the expectation of the motif occurring at each site in the UPC, which is a simple calculation based on the frequency of each amino acid (*f_a_*), and the total number of positions that a motif can occur (*N_m_*). By default, amino acid frequencies are calculated from the dataset, individually for each UPC, before any masking takes place. Additional options allow amino acid frequencies to be adjusted for masking, averaged over all UPCs, or read from a file.

For each defined position in a motif with d alternative (degenerate) amino acids, the probability of occurrence at any residue in the dataset (*p_i_*) is the sum of the frequencies for the possible amino acids at that position:
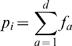
The probability *p_m_* of the whole motif starting at any residue is therefore the product of *p_i_* over all *L* positions in a motif: 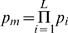
(Wildcard positions do not contribute to this value, as the probability of matching a wildcard is 1.0.). This defines the probability for each “Bernoulli trial” in the binomial distribution. What remains is to define appropriately the number of trials for the motif in the UPC. There are two features of the UPC that complicate estimation (for the probability calculation) of the number of positions that a motif might arise at: firstly, some but not all regions of the UPC proteins are related by evolution, and secondly, the particular pattern of masking may alter the number of positions available for motifs with a particular distribution of non-wildcard positions.

Because the proteins within a UPC are evolutionarily related, they do not contribute to the motif space searched by SLiMFinder in the same way as unrelated proteins, for which the motifs found would be independent. However, unless all the sequences are 100% identical, there are still more independent positions at which a given motif could occur than in any of the individual sequences within the UPC. The UPC must therefore be rescaled to represent its true contribution to the dataset. This is performed using the “Minimum Spanning Tree” (MST) correction used by SLiMDisc [8] to correct for evolutionary relationships. This MST value varies from 1 to *N*, where *N* is the number of proteins in the UPC. If all proteins are 100% identical the MST value is equal to 1 (and the UPC is exactly equivalent to a single sequence). As the proteins become more dissimilar, MST tends towards *N* (see SLiMDisc [8] for more details). This is converted into an “MST correction”, *M*, for the UPC by dividing the MST value by *N*. The total size of the UPC is therefore adjusted by multiplying *N_aa_* (the total number of unmasked residues in the UPC) by *M*. (This is equivalent to the mean number of amino acids per sequence in the UPC, multiplied by the MST-corrected size of the UPC.) SLiMFinder uses the largest GABLAM [8] ordered percentage identity between each pair of sequences to generate the distance matrix for MST calculations.

The distribution of masking influences the potential number of sites at which a motif can occur. For a dimer motif with a given wildcard length x, SLiMChance directly observes the frequency of positions in the dataset that could accommodate a dimer motif of that wildcard length. Then, for longer motifs, it estimates the frequency of potential sites as the product of the fraction of dimer sites for all the dimers that constitute the motif. This has the numerical advantage that the frequencies of dimer types are previously available from the SLiMBuild computation. The number of trials is then estimated as the possible number of positions at which the motif could start (*N_m_*). *N_m_* is calculated empirically from the dataset. During dimer generation, the number of i-x-j dimers (Figure 2), *N_ixj_*, is counted for each wildcard length *x* (where neither *i* nor *j* are masked). This is converted into the fraction of unmasked residues that start with a dimer of wildcard-length *x*, *D_x_*, calculated as a proportion of the unmasked positions (*N_aa_*) in the UPC. 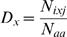

*N_m_*, the number of positions at which a motif may potentially occur is then calculated from the product of the motif's component dimer frequencies and the MST-adjusted number of unmasked residues in the UPC: 
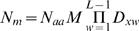
where *M* is the MST correction for that UPC, *L* is the length (no. of positions) of the motif and *D_xw_* is the dimer frequency for that wildcard length *x* at wildcard position *w*. (For flexible-length wildcards, this is the mean dimer frequency of the length variants at *w*.)

If there are wildcard length variants, each length variant has a chance of occurring and so this effectively increases the number of possible motif positions via a simple multiplication, where *x_j_* is the number of wildcard variants at wildcard position *j*: 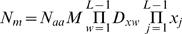
It could be argued that this multiplier should apply to the probability of the motif at each position, rather than the number of motif positions. (In reality, each motif “position” is a starting residue. Obviously, there cannot be more starting residues than the length of the sequence, whereas this multiplication implies that there can be.) The reason for applying the correction to *N_m_*, however, is that this value has no upper bound for the binomial calculation. The probability *p_m_*, in contrast, must be ≤1.0, whereas the multiplier for numerous variable-length wildcards could cause it to exceed 1.0.

The probability of 1+ occurrences of the motif in the UPC is calculated using the binomial: 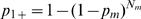



### SLiMChance probabilities per dataset

The individual *p_1+_* values are then used to calculate the motif probability for the entire dataset, *p*, where *N_U_* is the number of UPCs in the dataset and *K_U_* is the number of UPC containing the motif. Again, this is calculated using the binomial, where *p_u_* is the mean *p_1+_* value for each UPC: 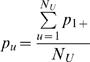


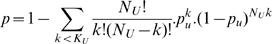



### SLiMChance significance values

The probability calculated above is the estimated probability of seeing a given motif with its observed support (or greater) given the dataset. However, the calculations implicitly assume that the motif was defined before anything was known about the dataset. In reality, SLiMFinder is looking for all possible motifs and only actually returning those at the “top end of the distribution”, *i.e.* the over-represented motifs. In reality, each motif in the “motif space” searched has a chance of being stochastically over-represented, so it is important to adjust for this and establish a significance value for each motif.

The *a priori* probability of each motif in motif space being over-represented with a probability *p* is itself (perhaps obviously) *p*. Because SLiMBuild generates motifs using a maximum wildcard spacer length, *X*, it is possible to calculate exactly the size of the motif space, *B_L_*, for each length of motif *L*: 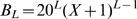
The significance of a motif (*Sig*) with occurrence probability *p* can therefore be calculated using the binomial distribution as the probability of getting one or more successes given *B_L_* trials of probability *p*. 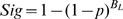

*Sig* ranges from zero to one and can be thought of as a true *p*-value. Because different lengths of motifs are not independent of each other, *Sig* is calculated independently for each number of defined positions. The motif space calculation only calculates the number of fixed-position motifs in the search space. Allowing ambiguities obviously increases the size of the search space and very relaxed ambiguous searches may need to use a more stringent *p*-value accordingly.

### SLiMFinder Output

The main output for SLiMFinder is a delimited text file containing the list of motifs that meet the user-specified threshold for corrected significance (*Sig*). These are ranked according to their significance. Each line also contains a number of dataset-specific fields, allowing multiple datasets to be run and analysed together. Additional outputs that assist the visualisation and interpretation of interesting results are explained in detail in the SLiMFinder manual, available at the website. Other options include a TEIRESIAS-style output of all motifs generated by SLiMBuild, allowing SLiMFinder to be used as a direct replacement for TEIRESIAS for other SLiM discovery tools.

### Systems

All SLiMFinder runs were performed on an Intel(R) Xeon(TM) dual 3.20GHz processor with 3Gb RAM. SLiMFinder and its constituent algorithms were run using Python 2.4.3.

### Disorder prediction

For analyses presented in this paper, IUPRED [11] was used to predict intrinsically unordered regions, using the “short” setting and a threshold of 0.2.

### Human genomic protein dataset

The EnsEMBL [12] human genome V.41 known and novel protein sequences were downloaded and used to generate a comprehensive, non-redundant sequence dataset containing one protein per gene. If a gene mapped to a SwissProt [13] sequence, and one of the peptides mapped to that gene had an identical sequence to the SwissProt entry then that peptide was used; in all other cases, the longest peptide was used. Sequences themselves were taken directly from the EnsEMBL. In total, this dataset consisted of 23,224 protein sequences, including 14,694 that mapped onto SwissProt entries.

### Random test data

To test SLiMFinder function on a range of random data with different levels of realism, three types of random data were generated: (1) Randomly generated sequences using uniform amino acid frequencies; (2) Randomly generated sequences using amino acid frequencies from the Human genomic protein dataset; (3) Randomly selected proteins from the Human genomic protein dataset. The mean length of a protein sequence in the human protein dataset was 487.4 amino acids. Random sequences were therefore generated from a random length distribution ranging from 200 to 800 amino acids, with a mean length of 500 amino acids. For each type of random data, ten replicates of each of twenty-five datasets sizes were generated: 3, 4, 5, 6, 7, 8, 9, 10, 12, 14, 16, 18, 21, 24, 27, 30, 35, 40, 45, 50, 60, 70, 80, 90 and 100 proteins. This produced 250 datasets for each type of random data.

### ELM benchmarking datasets

The best resource for biologically validated SLiMs is currently the Eukaryotic Linear Motif (ELM) database [4], which contains information for over a hundred known motifs, including example occurrences for many. (Other resources, such a Minimotif Miner [14], contain more motifs but have considerably less annotation.) ELM data has been used as a benchmark for previous SLiM discovery software [5], [8]. The benchmark dataset consisted of seventeen ELMs for which there were at least three annotated occurrences in *unrelated* proteins (Table 1). Each ELM dataset consisted of all the proteins with annotated occurrences from the ELM website (Jan 2007). At first glance, this seems like an “easy” test set, as every protein in the dataset contains the known motif. In reality, however, the motifs are often degenerate and different proteins will contain different variants, and so the re-discovery of the known motifs is far from a foregone conclusion [5], [8]. As ELM represents the most comprehensive resource of validated SLiM occurrences available, it is still the best benchmarking dataset for SLiM discovery validation.

**Table 1:** ELM benchmarking results sorted by significance of returned motifs.

ELM	N^a^	SLiMFinder^b^	Sig^b^	SLiMDisc^c^	DILIMOT^c^
TRG_ER_KDEL_1 [KRH][DENQ]EL	12 (10)	K.{0,2}DEL$ (1)	0.000	KDEL (1)	DEL (1)
LIG_Dynein_DLC8_1 [KR].TQT	4 (4)	S..K.TQT (1)	3.9×10^−6^	S..K.TQT (1)	TQT (1)
LIG_PCNA Q..[ILM]..[FHM][FHM]	13 (9)	[IL].S[FH]F (1)	4.3×10^−6^	Q..L..F (36)	Q.....FF (1)
MOD_SUMO [VILAFP]K.[EDNGP]	29 (19)	[FIV]K.E (1)	2.0×10^−5^	IK.E (2)	IKQE (1)
LIG_SH3_2 P..P.[KR]	9 (8)	P..P.R.{0,1}P (1)	0.004	PP.P (1)	PP..P.R (1)
LIG_CYCLIN_1 [RK].L.{0–1}[FYLIVMP]	22 (15)	RR.{0,1}L.{0,1}F (1)	0.005	KKL (7)	-
LIG_CtBP P.[DEN]L[VAST]	26 (12)	P[ILM]DL (1)	0.016	P.DL (1)	P.DLS (1)
LIG_AP_GAE_1 [DE][DES].[F].[DE][LVIMFD]	8 (5)	D.F..F.S..P (1)	0.40	D.F.DF.S (1)	F.DF.S (1)
LIG_14-3-3_3 [RHK][STALV].[ST].[PEDSIF]	6 (6)	S.P.S.T.P (3)	0.89	S.S.P (5)	S.SVS (2)
LIG_RB [LI].C.[DE]	25 (23)	L.C.E (6)	0.91	L.C.E (1)	L.C.E (1)
LIG_Clathr_ClatBox_1 L[ILM].[ILMF][DE]	15 (9)	L.{1,2}DL.{0,2}D (12)	0.93	L.DL (1)	L.DL (1)
LIG_14-3-3_1 R[FSWY].S.P	4 (3)	RS.S.P (3)	1.00	RS.S.P (1)	R.R..S (4)
LIG_RGD RGD	15 (7)	R.D.V (7)	1.00	RGD (1)	-
LIG_HP1_1 P.V.[LM]	6 (5)	-	-	P.V.L (1)	P.V.L (4)
LIG_NRBOX L..LL	9 (9)	-	-	L..LL (10)	-
MOD_N-GLC_2 N.C	5 (4)	-	-	-	-
TRG_LysEnd_APsAcLL_1 [DER]...L[LVI]	10 (9)	-	-	E…LL (27)	D.R.L (7)

a Number of proteins in dataset. Number of UPC is given in brackets.

b The most significant motif returned by SLiMFinder that matched the ELM, with its significance score. The rank of the motif is given in brackets. No pattern indicates that the top 100 motifs did not match the ELM.

c The top-ranked motif returned by SLiMDisc or DILIMOT (default parameters; predicted globular domains masked out) that match the ELM. The rank is given in brackets.

## Results

### SLiMFinder performance on random data

**Figure 4:**
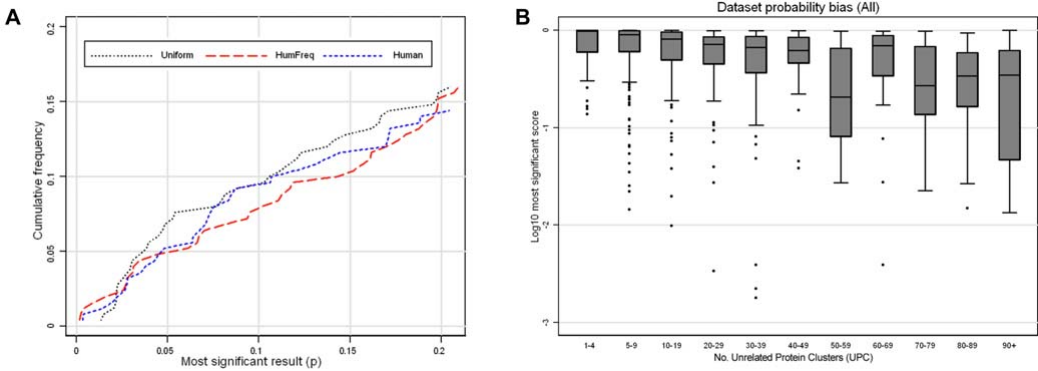
SLiMFinder results on random datasets. A. Cumulative frequency of the most significant motifs returned by SLiMFinder for random datasets. Very little difference is observed between datasets produced using human amino acid frequencies and datasets of actual human protein sequences, implying that there is little or no bias introduced by regional compositional biases within real protein sequences. B. Box plots of most significant results returned by all random datasets for different dataset sizes (UPC). Although there is a slight trend for larger datasets to return smaller p-values, the difference is primarily restricted to the non-significant motifs. Variation between datasets of the same size is considerably greater than variation between different sized datasets.

Before considering the performance of SLiMFinder on datasets of real biological interest, it is useful to assess its performance on random datasets. We looked at the most significant motif returned by each of 750 random datasets. The false positive rates for SLiMFinder are very similar, at any given significance threshold, for each type of random data (Figure 4A). Moreover, random data matches the calculated expectation quite closely, with approximately 10% of datasets yielding a significance of 0.1 or lower and 1% of datasets yielding a significance of 0.01 or lower. Although this relationship begins to deviate as the p-value increases, this is not of concern as these deviations occur within the non-significant portion of the data and will therefore not impact on results. Differences between different types of random data are minimal. Underlying complexities in amino acid distributions for real protein sequences, therefore, do not seem to strongly violate the underlying assumptions of the model.

It is also of interest to ask how the program scales with dataset size in terms of the results returned. SLiMDisc [8], for example, scales very poorly with dataset size: the number of motifs returned–and the scores of returned motifs–increases substantially. Although SLiMFinder shows some bias, the significance of the most significant motif returned from each dataset is not strongly dependent on dataset size (Figure 4B).

### SLiMFinder performance on ELM benchmark data

Seven of the seventeen ELM datasets yield significant motifs (*p*<0.05) that are variants of the true ELM (Table 1). This is not simply a reflection of how over-represented the true ELM is in the dataset, however. For the top three results, the true ELM is indeed “significant” but the remaining four ELMs that are found are not, as defined by ELM, particularly over-represented (data not shown). Instead, variants of the motif are discovered that *are* over-represented. These do not match the ELM exactly but the same is also true for the existing alternative SLiM discovery methods, SLiMDisc [8] and DILIMOT [7]. The SLiMChance score can therefore be seen as a complementary method to those previously implemented; it successfully returns motifs that the earlier methods did not, while failing to successfully identify several motifs as significant that SLiMDisc and/or DILIMOT returned. Indeed, even when SLiMFinder succeeds for the same datasets as DILIMOT and/or SLiMDisc, it generally returns a different motif variant: only two of the SLiMDisc/DILIMOT motifs would be classed as “significant” by SLiMChance (data not shown). SLiMFinder motifs tend to be longer and include more defined positions of the known ELM than motifs returned by either SLiMDisc or DILIMOT (Table 1). None of the seventeen datasets returned significant motifs that were *not* variants of the true ELM, supporting the evidence from random datasets that the SLiMChance significance exhibits high specificity.

So why did SLiMFinder fail for these additional ten motifs? Several of the datasets are quite small and yet the ELM itself is quite degenerate. The signal present in the dataset might therefore simply be too weak to detect regardless of the method. For four ELMs (LIG_14-3-3_3, LIG_NRBOX, MOD_N-GLC_2 and TRG_LysEnd_APsAcLL_1), none of the SLiM discovery methods returned a variant of the ELM as the top ranked result. For three others (LIG_14-3-3_1, LIG_HP1_1 and LIG_RGD) SLiMDisc returned the motif as the top rank but DILIMOT did not. Together, these account for 70% of failures. Importantly, pre-processing of the dataset can also impact on results. While it has been observed that SLiMs *tend* to occur in disordered regions [4], [6], masking UniProt “Domain” features and predicted disordered regions may mask out some true motifs. This certainly seems to be the case for LIG_RB. When disorder masking is switched off, LIG_RB returns the true ELM variant L.C.E as the most significant result, with a significance of 1.6×10^−10^ (data not shown). This highlights the need for considering carefully how to mask sequences (or not) prior to searching. In some situations, it will make sense to carry out searches both with and without masking. The remaining motifs are probably missed because their amino acid composition makes them highly likely to occur by chance (*e.g*. LIG_Clathr_ClatBox_1 is leucine-rich (the most common amino acid) and has a high degree of degeneracy) or the dataset is too small to achieve a likelihood value that survives the correction for motif space (*e.g.* LIG_14-3-3_1 is a “strong” motif but, with only three UPC in the dataset, there is simply not enough statistical power for it to be detected). In contrast, SLiMDisc is able to return these motifs as it does not depend on over-representation versus random expectation but instead relies solely on over-representation versus other motifs in the dataset. SLiMFinder therefore complements the capabilities of SLiMDisc, which remains useful for smaller datasets.

### Improved SLiMBuild amino acid ambiguity

One of the major improvements of SLiMFinder over SLiMDisc and DILIMOT is the way that amino acid ambiguity is incorporated. DILIMOT does not make use of ambiguity at all. SLiMDisc does have the option for including ambiguity but caution is advised, as it tends to increase substantially the return of false positives without much improvement in the motifs returned [8]. Ambiguity in SLiMFinder, however, does not introduce any false positives for the ELM benchmark dataset. Furthermore, the sensitivity of searches is increased by incorporating ambiguity. Of the seven ELMs yielding significant motifs, two fail to return significantly over-represented motifs without amino acid ambiguity.

This is exemplified by the LIG_PCNA motif, which returns 13 ambiguous variations of the defined ELM (data not shown), the third of which (Q..[IL].SFF) covers all defined positions of the ELM. Another feature of SLiMFinder is that it attempts to reduce the complexity of the output by grouping motifs into “clouds”. These clouds are generated in a pairwise fashion; each pair of motifs is considered in turn and if they share at least two defined positions in at least two occurrences (*i.e.* the same residue in the same protein), they are put together in the same cloud. Because “true” motifs are often short and/or degenerate, SLiMFinder will generally return a variant of the motif, often with additional defined residues (Table 1). This is presumably because the over-represented “core” of the motif increases the likelihood of an extended pattern (that includes the core) also appearing to be over-represented: this may be just chance, or may reflect additional genuine but weaker sequence features around the motif. By grouping motifs together in this way, the user can achieve a better sense of which residues in the motif are most important.

### Flexible wildcards

One of the innovations of SLiMBuild over TEIRESIAS [9], which is used to generate motifs for both SLiMDisc and DILIMOT, is its ability to return motifs with flexible-length wildcards. Although only a limited number of known ELMs have annotated flexible-length wildcards, their incorporation can increase the accuracy of discovery. The cyclin ligand motif LIG_CYCLIN_1 ([RK].L.{0,1}[FYLIVMP]), for example, is returned very well by SLiMFinder (RR.{0,1}L.{0,1}F) while SLiMDisc (KKL) and DILIMOT (None) struggle to return an accurate descriptor (Table 1).

### Combined ambiguity

In principle, SLiMFinder can return motifs with combined amino acid and wildcard ambiguities. In practice, however, this creates very long runtimes with little or no improvement in results, and is not recommended. (By default, SLiMFinder will return motifs with flexible wildcards and motifs with amino acid ambiguities but not motifs with both together.) For the ELM test dataset, no motif definitions were improved by combining both ambiguities during the SLiMBuild generation of motifs (data not shown). However, it is plausible that motif definitions may be improved by manually combining several motifs with different ambiguities from the same “motif cloud”.

### Additional of sequence termini characters

The additional of sequence termini characters (^ for the N-terminal and $ for the C-terminal) is a simple improvement that can help identify terminal motifs, such as the TRG_ER_KDEL_1 Golgi-to-ER retrieving signal. Although the KDEL motif alone is found as highly significant by SLiMChance (1.29×10^−4^; data not shown), the addition of the C-terminus symbol increases the significance by over twenty orders of magnitude. It is envisaged that for more borderline terminal motifs, the extra significance given by the proximity to the termini could be vital in identifying such motifs.

### SLiMBuild versus TEIRESIAS runtimes

The primary motivation behind SLiMFinder was to improve the results of *ab initio* SLiM discovery by generating better motif descriptors and attaching a significance value to results. It is important, however, that these improvements in performance are not achieved at the cost of realistic runtimes. The best predictor of runtimes for random datasets was the number of amino acids in the dataset (data not shown). Although SLiMFinder runtimes do appear to increase exponentially with increasing dataset size, the slope of the curve is very shallow and none of the test datasets took more than an hour to run on a single 3.2GHz processor (Figure S2A). Indeed, all 750 test datasets could be run on a single machine in under 86 hours, making SLiMFinder very feasible for large scale analyses. In addition, the explicit treatment of the dataset to return convergently evolved motifs maintains manageable run-times as the degree of relatedness of the input dataset increases (Figure S2B). TEIRESIAS runtimes, in contrast, increase as the number of related proteins increases. This problem is magnified by use of ambiguity, in which case even small datasets can take several hours to run with TEIRESIAS. For an arbitrary dataset of twelve unrelated proteins that interact with AAA-domain proteins, for example, adding the default SLiMBuild equivalencies (AGS, ILMVF, FYW, FYH, KRH and DE) increased the runtime of TEIRESIAS by more than three orders of magnitude from 30 seconds to over 13 hours. For the same equivalency groups on the same dataset, the runtime of SLiMBuild was increased by approx 25% from 54 seconds to 68 seconds.

### Example application 1: 14-3-3 interaction datasets

SLiMFinder performs with reasonable success on the ELM test data but it is of interest to see how it performs in what could be considered the more challenging case of real, often noisy, datasets. Two of the ELM datasets that “failed” were 14-3-3 ligand datasets. This failure could largely be attributed to the small dataset sizes of the test sets, with only 3 unrelated proteins for LIG_14-3-3_1 and 6 unrelated proteins for LIG_14-3-3_3. Increasing the dataset size, even if this introduces some “noisy” sequences that do not contain the motif, can allow such a motif to be returned. Furthermore, the ELM LIG_14-3-3_2 was not included in the test dataset due to the small number of annotated occurrences on the ELM website.

We therefore sought to find 14-3-3 ligand motifs in the larger, but noisier, data available in the HPRD database [15]. Humans have seven 14-3-3 isoforms, each with interaction data available in HPRD. SLiMFinder was run on each dataset and motifs with at least three unrelated occurrences and significance of 0.05 or less returned (Table 2). Regions predicted to be ordered with IUPred [11] were masked out. Consistent with the predicted high stringency of SLiMFinder, two of the datasets yielded no significant results. Of those that did, three returned 14-3-3-like motifs (R..S.P.L, GR.[ST]..P and FR..[ST].S). Furthermore, two datasets returned probable SH3-binding P..P motifs. Two datasets returned N-terminal motifs, which may represent common N-terminal target peptide signals rather than ligand-binding motifs. Only two additional motifs were returned, KE..K and Y.C.PG.L, neither of which are known SLiMs. These motifs may represent novel findings relating to 14-3-3 binding or function, although given the low significance of these motifs (0.01<*p*<0.05) we cannot rule out the possibility that they are false positives.

**Table 2:** SLiMFinder results for 14-3-3 interaction datasets from HRPD.

Isoform^a^	N^b^	Pattern^c^	Coverage^d^
Beta/Alpha (YWHAB)	220 (55)	-	
Epsilon (YWHAE)	117 (34)	R..S.P..L *	40.0%
Eta (YWHAH)	83 (27)	GR.[ST]..P *	37.0%
Gamma (YHWAG)	383 (101)	^.[AS][AGS] ***	40.6%
		KE..K *	35.6%
Sigma (SFN)	48 (21)	-	0.0%
Theta/Tau (YWHAQ)	132 (42)	P..P..P *	66.7%
Zeta/Delta (YWHAZ)	190 (58)	[AGS]..P..P..P ***	48.3%
		^.[AGS][GS] **	27.6%
		FR..[ST].S **	19.0%
		[ST]P.[ST]P *	34.5%
		Y.C.PG.L *	6.9%

a 14-3-3 isoform. HGNC gene symbol given in brackets.

b Number of proteins in dataset. Number of UPC is given in brackets.

c The most significant motif of each “cloud” returned by SLiMFinder.

* *p*<0.05,

** *p*<0.01,

*** *p*<0.001.

d The percentage of the dataset's UPC covered by occurrences of returned motifs in the same motif cloud.

### Example application 2: Endoplasmic reticulum membrane targeting signals

In addition to the TRG_ER_KDEL_1 motif, ELM contains two more endoplasmic reticulum targeting SLiMs, both of which also lie at/near the termini of ER membrane proteins: TRG_ER_diArg_1 (^M[DAL][VNI]R[RK] or ^M[HL]RR) and TRG_ER_diLys_1 (K.{0,1}K.{2,3}$)[4]. The KDEL motif, in contrast, is found in soluble proteins [4]. We took the Gene Ontology cellular component category that identified endoplasmic reticulum proteins (GO:0005783 “endoplasmic reticulum”) and extracted all sequences matching this categories from six taxonomically diverse EnsEMBL genomes (*C elegans*, Chicken, Drosophila, Human, Yeast and Zebrafish) [12]. SLiMFinder was run on each dataset and motifs with a significance of 0.05 or less returned. To enrich for targeting SLiMs, we restricted analysis to the 20 amino acids at each terminus. The TRG_ER_KDEL_1 motif was returned by five out of six datasets, while the TRG_ER_diLys_1 motif was returned by three (Table 3). The TRG_ER_diArg_1 motif was not returned. On closer inspection, this motif occurs few or no times in each dataset. In addition to these known motifs, a number of novel motifs were returned. The most interesting of these were the L.FL.{0,1}L and, overlapping, [FV].L.L motifs, which were found in five out of six *C. elegans* UPC and were the only significant motifs returned from this dataset. The top-ranked motif for the human dataset was ^.A..G, which occurred in 28 unrelated proteins. This is the result of an over-representation of alanine at the second position in these proteins, which may be indicative of a shared N-terminal target peptide sequence.

**Table 3:** Results of SLiMFinder analysis performed on human and yeast ER proteins.

Species	N^a^	Top Rank^b^	diArg^b^	diLys^b^	KDEL^b^
*C. elegans*	10 (6)	L.FL.{0,1}L **	-	-	-
Chicken	40 (30)	DEL$ *	-	-	DEL$ *
Drosophila	168 (69)	[HK].EL$ ***	-	[KR]K..$ *	[HK].EL$ ***
Human	618 (346)	^.A..G ***	-	KK..$ ***	DEL$ ***
Yeast	318 (249)	HDEL$ ***	-	KK.N$ ***	HDEL$ ***
Zebrafish	76 (42)	[HK].EL$ ***	-	-	[HK].EL$ ***

a Number of proteins in dataset. Number of UPC is given in brackets.

b The most significant motif returned by SLiMFinder.

* *p*<0.05,

** *p*<0.01,

*** *p*<0.001.

c The most significant motif returned by SLiMFinder that matched the known ER ELMs. KDEL = LIG_ER_KDEL_1; diLys = LIG_ER_diLys_1

### Example application 3: HBV phage display

Hepatitus B virus (HBV) is thought to infect human hepatocytes via attachment of the viral envelope protein's PreS domain with a specific cell surface receptor, the identity of which is unknown [16]. Deng *et al.* sought to identify novel binding partners of the PreS domain using phage display. From a random phage display library of 12mer peptides, they isolated 13 phages with specific PreS-binding activity, which in turn represented nine different peptide sequences. The authors noted a high frequency of tryptophan residues and manually determined a putative consensus sequence of WT.WW from a multiple sequence alignment of the peptides. This sequence was itself able to bind HBV particles. By searching candidate interactors with a slightly degenerate [FW]T.W[FW] motif (using BLAST), Deng *et al.* successfully identified a novel receptor protein, lipoprotein lipase (LPL), which also bound HBV.

This work is an excellent example of how phage display can be used to identify a novel SLiM mediating a protein-protein interaction. However, given the short length of phage display peptides, using multiple sequence alignment to identify the shared motif(s) is not ideal and an alignment-free method may be less susceptible to bias. In addition to potential alignment errors, choice of consensus is a subjective human decision. We applied SLiMFinder to the nine 12mer peptides that bound PreS. Using amino acid frequencies from the input dataset, unsurprisingly, returned no significant motifs. This is because the dominating tryptophans are so prevalent that they make tryptophan-containing motifs statistically highly probable. The reality, however, is that these peptides were selected from a population of sequences with a very different amino acid composition. We therefore replaced the dataset amino acid frequencies with amino acid frequencies derived from the whole human genome. As expected, all significant motifs featured tryptophan, with the top ranked motif, W.{0,2}W being highly significant (*p* = 8.2×10^−15^). A three amino acid variant, W.{0,2}WW, which is similar but subtly different to the consensus of Deng *et al.*, was also significant (*p* = 1.5×10^−6^). In the active LPL protein, it is this W..WW motif that is conserved, while the consensus “T” is not a threonine in LPL [16]. The manually generated WT.WW motif was not significantly over-represented (*p* = 1.00). The degenerate [FW]T.W[FW] motif is significantly over-represented (*p* = 0.016) but was not returned as the appropriate component variants do not each occur in two or more sequences.

Although no common motifs have been found to date, we also investigated the possibility that proteins previously reported to bind the key region of PreS shared motifs with the phage display peptides. Interleukin 6 [17] and Serpin B3 (also known as Squamous cell carcinoma antigen 1) [18] were therefore added to the peptide sequences and SLiMFinder re-run and results analysed for motifs occurring in at least one of the full-length human proteins. The third-ranked motif, [FW]W (*p* = 5.3×10^−7^) was found in Serpin B3. This motif was also returned third from the peptides alone. This motif occurred in seven of the nine peptides, Serpin B3 and the LPL protein. A common mode of action for PreS-binding of Serpin B3 and LPL cannot be ruled out, therefore. The [FW]W is highly conserved in both Serpin B3 and LPL orthologues (data not shown), although the significance of this is limited unless HBV is shown to infect other species via a similar mechanism.

In this example, the motif was so striking that, in reality, use of SLiMFinder did not add much value to the manual interpretation, except for a degree of statistical support for identified motif. In other situations, however, we can envisage the impact being much more significant. If the motif is more cryptic, then alignment-based manual inspection is much less likely to succeed. Perhaps more importantly, use of SLiMFinder in this context produces repeatable results and is therefore suitable for being scaled up to analyse and compare multiple datasets in a more objective fashion.

### Conclusion

The full potential of SLiMs, both as explanations for biological phenomena and as experimental tools in molecular biology, is only just being unlocked [1]. To meet this potential, there is a requirement for both improvements in technologies to identify protein-protein ligand interactions and in the methods to identify SLiMs from the results of these technologies. Existing methods can be effective but suffer from low specificity of predictions, which can reduce the willingness of experimental biologists to act on the results. SLiMFinder is a novel algorithm building on, and extending from, the approaches of SLiMDisc [8] and DILIMOT [7] to improve both the nature of the motifs returned and confidence in the predictions. SLiMBuild improves the type of motif returned through improved incorporation of ambiguity, introduction of flexible-length wildcard “gaps” and more control over the composition and length of motifs. The SLiMBuild approach has a number of advantages for SLiM discovery over TEIRESIAS [9] and would therefore make a worthwhile replacement of TEIRESIAS for other SLiM discovery methods, such as DILIMOT [7] or SLiMDisc [8]. Furthermore, the way that motifs are assembled by SLiMBuild makes it possible to make accurate estimates of the motif-space searched. SLiMChance takes advantage of this feature of SLiMBuild to combine accurate predictions with high stringency. This is the first practical application in this area that attempts to calculate a relevant significance value, and while the calculation is approximate, it provides a more useful guide for high-throughput analyses of many datasets. As the experimental techniques improve, and are applied more widely, it is hoped that the data available for SLiM detection will further increase the ability to identify new SLiMs with high reliability. Further refinements of the statistics will in turn give experimental biologists more faith in the results, encouraging more generation of high quality datasets specifically for motif discovery, such as the use of phage display peptides [16] and large scale interactome motif studies [5].

## Supporting Information

Figure S1 Anatomy of a SLiM. Definitions of different properties of SLiM have been marked on the example ELM, LIG_CYCLIN_1. This motif has three defined positions (one fixed and two degenerate) and two wildcard spacers (one fixed, one flexible-length) for a total length of 4-5aa.
(0.12 MB TIF)

Figure S2 SLiMFinder runtimes. A. SLiMFinder runtimes against dataset size. As expected, SLiMFinder takes longer to run with increasing dataset size. However, for typical dataset sizes of up to 100 proteins, runtimes remain short enough to make high throughput analyses feasible, even on a single processor. B. SLiMBuild run-times compared to TEIRESIAS runtimes for 12 proteins that interact with AAA domain-containing proteins from HPRD. Each dataset contains one or more related proteins from the ATP-binding cassette (ABC) family of proteins plus a number of unrelated proteins to make the total twelve. As the number of relationships increases, so the TEIRESIAS (square, dotted lines) runtime increases due to all the shared patterns between related sequences. SLiMBuild (triangles, solid lines), in contrast, ignores these patterns and so runtimes remain reasonably constant.
(0.90 MB TIF)
