# Supplementary figures and images for: SLiMFinder: A Probabilistic Method for Identifying Over-Represented, Convergently Evolved, Short Linear Motifs in Proteins
Source: PLoS One. 2007 Oct 3;2(10):e967. doi: 10.1371/journal.pone.0000967 (PMC1989135; doi:10.1371/journal.pone.0000967)

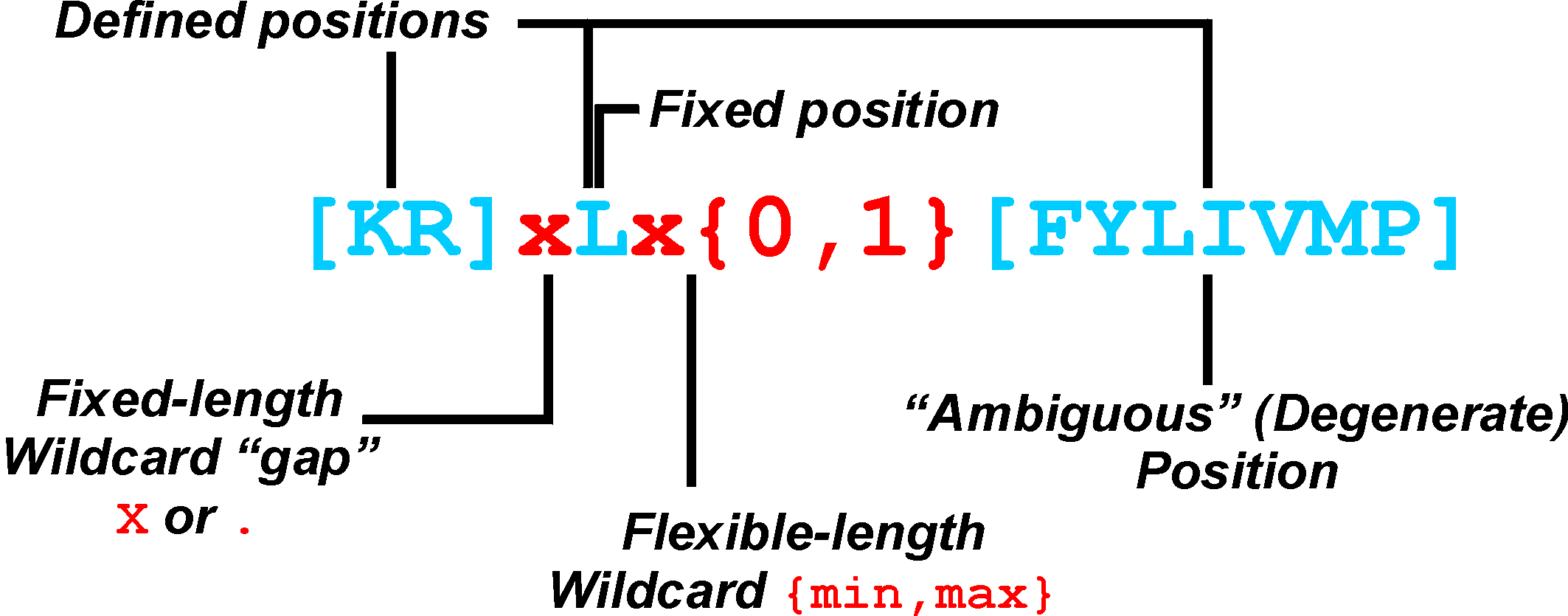

Supplement: Figure S1 — Anatomy of a SLiM. Definitions of different properties of SLiM have been marked on the example ELM, LIG_CYCLIN_1. This motif has three defined positions (one fixed and two degenerate) and two wildcard spacers (one fixed, one flexible-length) for a total length of 4-5aa. (0.12 MB TIF) [file pone.0000967.s001.tif]

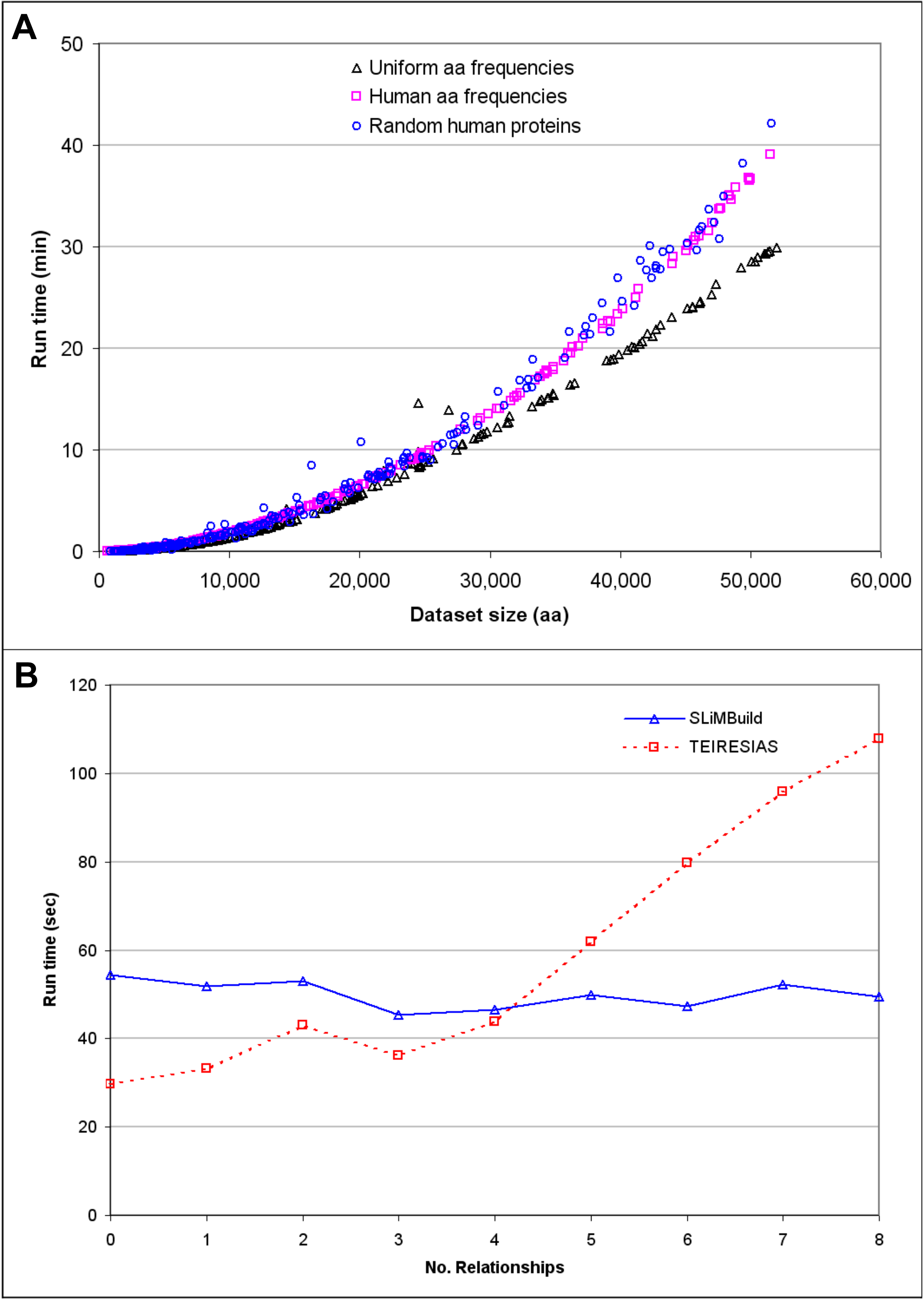

Supplement: Figure S2 — SLiMFinder runtimes. A. SLiMFinder runtimes against dataset size. As expected, SLiMFinder takes longer to run with increasing dataset size. However, for typical dataset sizes of up to 100 proteins, runtimes remain short enough to make high throughput analyses feasible, even on a single processor. B. SLiMBuild run-times compared to TEIRESIAS runtimes for 12 proteins that interact with AAA domain-containing proteins from HPRD. Each dataset contains one or more related proteins from the ATP-binding cassette (ABC) family of proteins plus a number of unrelated proteins to make the total twelve. As the number of relationships increases, so the TEIRESIAS (square, dotted lines) runtime increases due to all the shared patterns between related sequences. SLiMBuild (triangles, solid lines), in contrast, ignores these patterns and so runtimes remain reasonably constant. (0.90 MB TIF) [file pone.0000967.s002.tif]
